# Evoked and spontaneous pain assessment during tooth pulp injury

**DOI:** 10.1038/s41598-020-59742-5

**Published:** 2020-02-17

**Authors:** Heather Lynn Rossi, Lily Pachanin See, William Foster, Saumitra Pitake, Jennifer Gibbs, Brian Schmidt, Claire H. Mitchell, Ishmail Abdus-Saboor

**Affiliations:** 1 Department of Biology, University of Pennsylvania, Philadelphia, PA USA; 2 Department of Endodontics, University of Pennsylvania, Philadelphia, PA USA; 3 Department of Endodontics, Harvard University, Boston, MA USA; 4 Department of Oral and Maxillofacial Surgery, New York University, New York, NY USA; 5 Department of Basic and Translational Science, University of Pennsylvania, Philadelphia, PA USA

**Keywords:** Neuroscience, Somatic system, Pain

## Abstract

Injury of the tooth pulp is excruciatingly painful and yet the receptors and neural circuit mechanisms that transmit this form of pain remain poorly defined in both the clinic and preclinical rodent models. Easily quantifiable behavioral assessment in the mouse orofacial area remains a major bottleneck in uncovering molecular mechanisms that govern inflammatory pain in the tooth. In this study we sought to address this problem using the Mouse Grimace Scale and a novel approach to the application of mechanical Von Frey hair stimuli. We use a dental pulp injury model that exposes the pulp to the outside environment, a procedure we have previously shown produces inflammation. Using RNAscope technology, we demonstrate an upregulation of genes that contribute to the pain state in the trigeminal ganglia of injured mice. We found that mice with dental pulp injury have greater Mouse Grimace Scores than sham within 24 hours of injury, suggestive of spontaneous pain. We developed a scoring system of mouse refusal to determine thresholds for mechanical stimulation of the face with Von Frey filaments. This method revealed that mice with a unilateral dental injury develop bilateral mechanical allodynia that is delayed relative to the onset of spontaneous pain. This work demonstrates that tooth pain can be quantified in freely behaving mice using approaches common for other types of pain assessment. Harnessing these assays in the orofacial area during gene manipulation should assist in uncovering mechanisms for tooth pulp inflammatory pain and other forms of trigeminal pain.

## Introduction

Pain from the infected tooth pulp (pulpitis) can be unrelenting and many patients report this form of pain as the most intense type of pain they have ever experienced^1^. Mechanical hypersensitivity of the tooth is associated with greater pain intensity ratings overall^2^. Prevailing treatment options for painful pulpitis consists of pulp or tooth removal, which can have lasting consequences for dental function and in some patients there may still be lingering pain^3,4^. Therefore, there is a critical need for development of new therapeutic approaches that alleviate tooth pain while leaving pulpal issue intact and avoiding complex dental procedures. Moreover, untreated ongoing inflammation of the pulp can lead to more widespread nociceptive hypersensitivity in trigeminal tissues, an issue further compounded in individuals who cannot afford proper dental treatment^5^. The era of new innovative approaches to treat tooth pain will be driven by an increase in our fundamental understanding of the genes and neural circuit pathways that drive tooth pain states, and this understanding is dependent on mouse models. However, we first need to establish feasible and objective behavioral paradigms that measure pain in the orofacial area in preclinical rodent models.

Clinically, mechanical hypersensitivity and spontaneous pain are particularly problematic for patients^2–4^, and there are common behavioral assessment tools for these in rodent models when the pain occurs elsewhere in the body outside of the face. To date, only a handful of studies using the tooth pulp injury model have examined mouse behavior, and these studies have not incorporated some of the common assays used to measure nociception^6–8^. The predominant assessment tool for mechanical pain measurement in rodents are reflexive withdrawal assays in which calibrated von Frey hair filaments (VFHs) are applied to the hind paw and the experimenter decides which filaments evoke paw withdrawal^9^. We have recently improved on this method by incorporating high-speed videography, statistical modeling, and machine learning to more objectively assess the mouse pain state following hind paw stimulation^10^. VFHs can also be applied to the face, but this presents more challenges because the animal’s attention is more engaged with the stimulus, as we have previously experienced^11^. However, recent elegant work in freely behaving mice used both VFH stimulation of the whisker pad and optogenetic activation of trigeminal nociceptors to uncover a craniofacial neural circuit for pain^12^, demonstrating feasibility of the VFH approach for the study of oral pain.

Another approach thought to measure spontaneous pain in rodents is a paradigm called the Mouse Grimace Scale (MGS), that interrogates facial expressions including the positioning of the mouse nose, cheek, ear, eye, and whiskers^13,14^. An advantage of the MGS over reflexive assays is that spontaneous pain resembles pain reports in the clinic and facial expressions are used in the clinic to measure pain in infants, although these assays are currently not as high-throughput in rodents as delivering stimuli and recording immediate responses. Together however, both reflexive and spontaneous measurements of pain-like behavior provide advantages in that they can be performed without anesthesia, invasive implants, or time intensive tasks performed by the animal that require long term learning and memory, which may mean that interpretation of the behavior may be confounded by factors outside of the animal’s pain level.

The primary goal of this study was to optimize the assessment of pain-like behavior in conscious, unrestrained mice following induction of pulpitis. We also sought to determine the onset and duration of these pain-like behaviors during the first six days of pulp exposure. Here we take advantage of existing assays, with some custom modifications, and adapt them for assessment of pain-like behaviors during tooth pulp inflammation. After morphologically confirming our tooth pulp injury model, we used RNAscope technology to determine the time course of changes in molecular mediators of nociception in the peripheral nervous system relative to behavioral changes. To the best of our knowledge, this is the first study using the MGS to evaluate nociception following dental injury, and our results revealed the occurrence of spontaneous pain-like behavior within the first day following dental injury. We also adapted previous facial Von Frey methods^15^ to evaluate mechanical sensitivity, relying on the published scoring scheme, as well as the animals’ willingness to put its head through a custom designed chamber with an adjustable opening for stimulation. Because the mice can decide if they want to expose their faces to the stimuli, we were also able to record the threshold in which mice are no longer inquisitive enough to tolerate facial stimuli, and this tolerance threshold was able to segregate injured versus sham mice. Interestingly, these two assays present a different time course following injury, indicating putative spontaneous pain early and throughout the 6-day observation period, while mechanical allodynia and stimulation intolerance is delayed. The fact that we note bilateral changes in the response to mechanical stimuli suggests that central sensitization is also occurring, but the relationship between changes we observed in the ganglia and central mechanisms have yet to be uncovered. Taken together, the behavioral assays we have defined here to assess pain-like behavior in the mouse should make it easier for researchers to adopt these approaches to aid in uncovering mechanisms for tooth pain.

## Results

### Morphological and gene expression changes twenty-four hours after tooth pulp exposure

**Figure 1:**
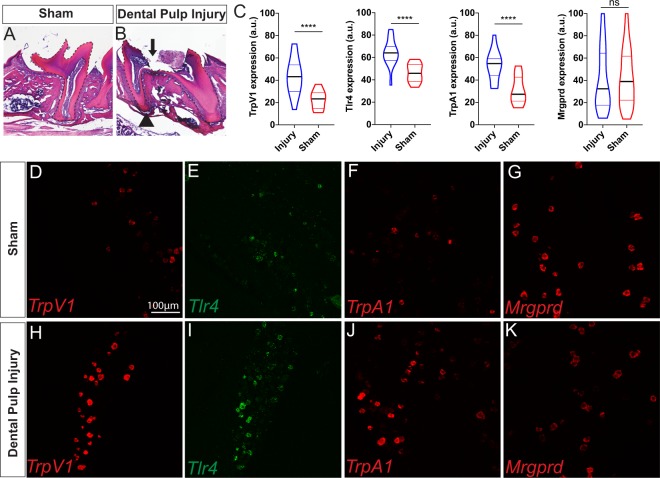
Changes in tooth morphology and trigeminal ganglia transcript levels 1 day following pulp injury. (**A**) Intact first maxillary molar from a sham animal and (**B**) injured maxillary molar with pulp exposure evident. Dotted outline marks the molar and the arrow indicates the exposure site. Healthy pulp is still evident on either side of the opening, and compacted foreign material (next to arrow) was present in the cavity. Arrowhead marks an area showing a potential periapical lesion typically seen in tooth infections. (**C**) Violin plot of quantified fluorescence intensity measured in arbitrary units (a.u.) defined in ImageJ for TrpV1, Tlr4, TrpA1, and Mrgprd in the trigeminal ganglia of injured (blue, n = 3 mice) or sham/naive (red, n = 3 mice) mice. (**D**–**G**) Representative images of RNAscope *in situ* hybridization following sham and (**H**–**K**) 1 day after dental pulp injury. **** is p < 0.0001 for an unpaired T-test.

In order to study inflammatory tooth pain, we used the dental pulp injury (DPI) model that we have previously described^6^ in which the dental pulp of one maxillary molar tooth is mechanically exposed using a dental drill, producing pulpitis. We began our first analyses 24 hours post-injury, and confirmed controlled removal of enamel and dentin and exposure of the pulp occurred in the molars of DPI-treated mice (Fig. 1A,B). Foreign material was microscopically present in the tooth cavity of all 3 injured molars, sometimes in contact with the pulp (material next to arrow in Fig. 1B), demonstrating that an exposed pulp collects materials from the mouse’s outside environment. Importantly, the pulp was still present and clearly exposed, and not yet necrotic, within the injury site at 24 hours (arrow Fig. 1B).

Next, we utilized RNAscope technology for a sensitive read-out of RNA levels in the trigeminal ganglion of genes implicated in both nociceptive and inflammatory responses. The trigeminal ganglion was examined as cell bodies of the primary afferent neurons that innervate the dental pulp reside there^16^. We chose to assess the Toll-like Receptor 4 (Tlr4), transient receptor potential channels vanilloid 1 and ankyrin 1 (Trpv1 and Trpa1), and the mas-related G protein coupled receptor D (Mrgprd), because all are found in neurons that innervate the dental pulp^7,17–21^ and could be involved in the development of either spontaneous or mechanical pain in the context of infection and injury. In particular, TLR4 is part of a larger class of receptors that recognize pathogen- and damage- associated molecular patterns (PAMPs and DAMPs)^22^, and has known interactions with both TRPV1 and TRPA1 in the context of dental injury^19,21,23,24^. A direct role for Mrgprd in dental injury-related pain has not been established, but is possible given its expression in dental afferents^17^ and its role in cutaneous mechanical pain perception^25^. We found that the PAMP/DAMP family member Tlr4 was upregulated in DPI-treated versus sham-treated mice (Fig. 1C,E,I), as were the associated nociceptive channels TrpV1 (Fig. 1C,D,H), and TrpA1 (Fig. 1C,F,J). However, we did not observe an increase in the mechanosensitive nociceptor marker Mrgprd at 24-hours following DPI treatment, suggesting that gene expression changes of this nociceptive membrane protein may not be driving the earliest phases of pain in the DPI model (Fig. 1C,G,K).

### Mouse grimace scale reveals presence of spontaneous pain beginning one day following pulp exposure

**Figure 2:**
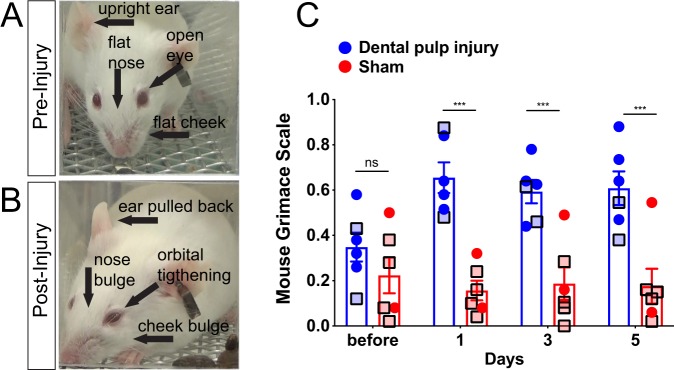
Mouse Grimace Scale following pulp exposure. (**A**) Before injury mice have low or no score for each of the action units, while after injury (**B**) prominent presence of the action units, as labeled on the example images from the same mouse. (**C**) We found a significant increase in the Mouse Grimace Score at all post-exposure time points captured, n = 6/treatment, Dental Pulp Injury (blue) and sham (red). Square shapes with black borders and lighter data points indicate females. *Indicates p < 0.0001 within the pulp exposed group before vs. after (Dunnett’s post-hoc).

To assess spontaneous pain-like behavior in freely behaving DPI-treated and sham-treated mice, we moved mice into clear custom-made chambers and recorded video of their faces. Still images were selected from these videos for assessment with the MGS (Fig. 2A). All mice, regardless of treatment exhibited very low MGS scores at baseline, which was not different between the assigned treatment groups and was not significantly affected by the sham treatment (Fig. 2). We found a significant increase in the MGS at all post-exposure time points captured (Fig. 2C, there was a significant effect of time, F_3,30_ = 5.776 p = 0.0031, treatment F_1,10_ = 18 p = 0.0017, and a significant interaction, F_3,30_ = 12.75 p < 0.0001). The MGS features that differed in the DPI-treated group versus sham-treated were the pulling back of the ears, nose and cheek bulging, as well as orbital tightening (Fig. 2B). These results are consistent with the previous report showing that MGS scores are highest for pain emanating from internal organs as opposed to the skin^13^, demonstrating that this assessment tool can be successfully co-opted for painful pulpitis. This finding also indicates that mice experience ongoing pain within the first day following pulp exposure that persists throughout the observation period.

### Mechanical allodynia in the face is fully developed by day 4 post pulp exposure and worsens

**Figure 3:**
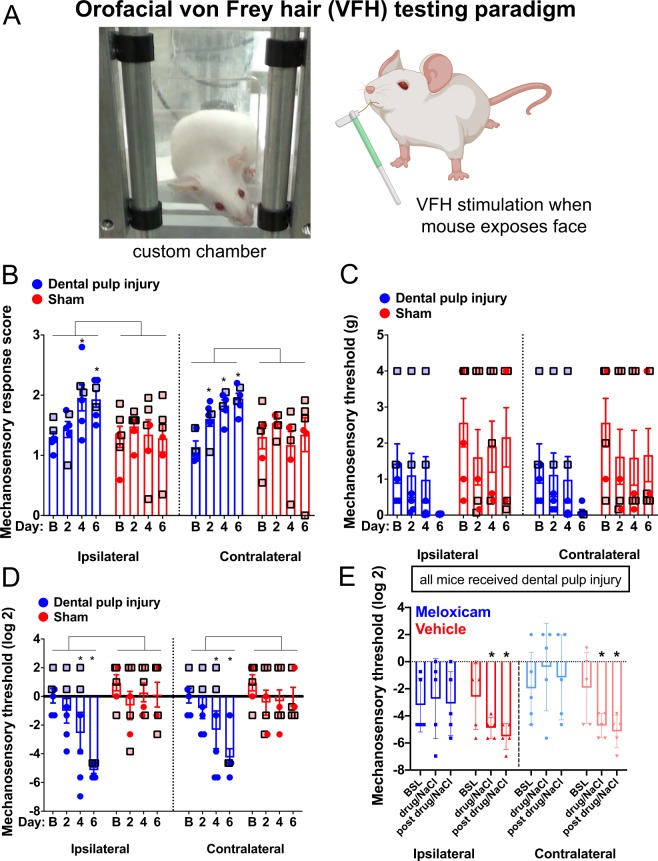
Facial Von Frey apparatus, mechanosensory response scores, and threshold changes following pulp exposure. (**A**) The mice were placed in the chamber for testing, which has adjustable openings that can be closed between tests and allows mice to put their head past the bars if they choose. When mice choose to expose their faces they are stimulated as depicted with a von Frey hair. Image created at Biorender.com. (**B**) After dental pulp injury (blue) there is a significant increase in mechanosensory response scores across all of the filaments tested, which is not observed in sham-treated mice (red). This is evident from day 4 onward on the ipsilateral side, and on day 2 onward on the contralateral side. (**C**) Raw thresholds are significantly different from normality according to the Shapiro-Wilks Test, but a decrease with time is apparent in the injured group not observed in sham mice. (**D**) After log transformation of threshold data to conform to normality, we find a significant decrease in threshold following pulp exposure on both sides, evident from day 4 and significantly lower than shams at day 6. *Indicates p < 0.05 for the indicated time point versus baseline (Dunnett’s post-hoc). Square shapes with black borders and lighter data points indicate females. (**E**) Saline treated mice had a significant decrease in their threshold at both time points following the injury, which did not occur in meloxicam treated mice. *Indicate p < 0.05 by Dunnett’s post-hoc test. All males used in panel E.

**Figure 4:**
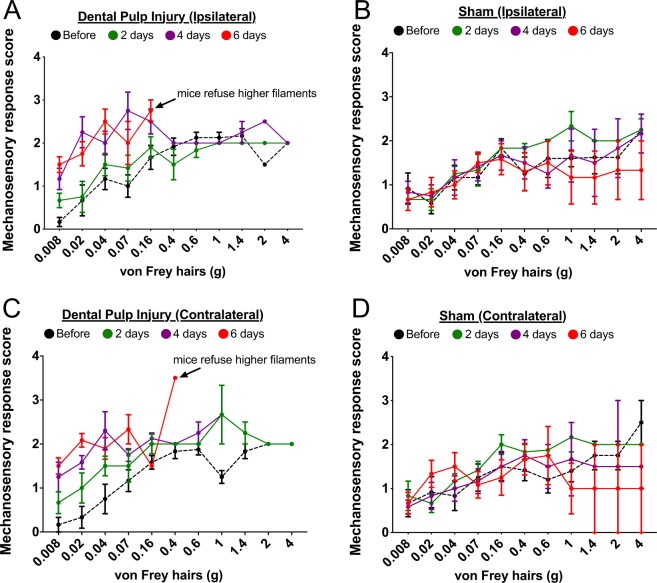
Mechanosensory response score by filament intensity and day post-injury or sham. (**A**) Ipsilateral and (**C**) contralateral response scores for low intensity filaments are increased at day 4 (purple) following injury, and by day 6 (red) mice met threshold criteria or refused filaments higher than 0.16 or 0.4 g. In contrast, (**B**) ipsilateral and (**D**) contralateral scores were similar across days post-sham procedure and low filament scores did not increase over time.

**Figure 5:**
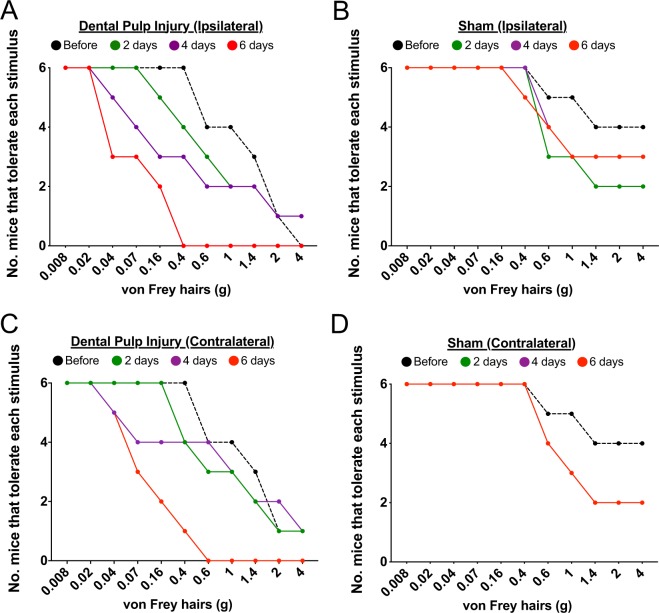
Loss of mouse participation by stimulus intensity (the break point) before and after injury or sham. On the (**A**) Ipsilateral and (**C**) contralateral sides, there is a progressive decrease in the number of injured mice willing to tolerate filaments higher than 0.4 g, such that by day 6 none of them progressed further than 0.16 g (ipsilateral) or 0.4 g (contralateral). In contrast, on the (**B**) ipsilateral and (**D**) contralateral sides the sham cohort consistently tolerated testing with lower intensity filaments and at least two mice tolerated all the filaments across all testing days.

To determine how mice respond to evoked stimuli, von Frey hair filaments were applied to the skin between the whisker pad and eye in DPI-treated and sham-treated mice and we scored the behavioral responses associated with the stimulus. Although our stimulus did not touch the tooth directly, hypersensitivity in the orofacial skin surrounding teeth, could indicate a more widespread trigeminal sensitization. Such hypersensitivity has been demonstrated in rats and is reminiscent of findings in the clinic when treatment complications arise^26,27^. Additionally, mice had to poke their heads through our custom-made chambers to allow the VFHs to make contact with the facial skin. Using this paradigm, we observed that unilateral exposure of tooth pulp on one molar changes response scores and thresholds for Von Frey stimulation on both sides of the face suggestive of mechanical allodynia (Figs. 3–5). The earliest significant change was an increase in response scores across all VFHs averaged together, at day 2 on the contralateral side (Fig. 3B, Contralateral: a significant effect of time: F_3,30_ = 6.43 p = 0.0017, a significant interaction: F_3,30_ = 7.69 p = 0.0006, but no significant effect of treatment alone: F_1,10_ = 2.272 p = 0.1626). By day 4, response scores are significantly increased in pulp-exposed mice on both sides, which persists on day 6 (Fig. 3B, Ipsilateral: significant effect of time: F_3,30_ = 3.302 p = 0.0336, no significant effect of treatment alone: F_1,10_ = 2.34 p = 0.1571, but significant interaction: F_3,30_ = 4.836 p = 0.0073). Overall this indicates that injured mice, but not sham-treated mice, exhibit a gradual increase in response scores that is most evident on day 4 and maximal on day 6.

We also examined the threshold where animals either scored a 3 or refused stimulation. Raw thresholds (Fig. 3C) were log-transformed to better conform to normality (Fig. 3D) and statistical analysis of transformed data indicated a significant decrease from baseline threshold on both ipsilateral and contralateral sides beginning at day 4 post pulp exposure. On day 6, the thresholds of pulp exposed mice were significantly lower than shams (a significant effect of time: ipsilateral - F_3,30_ = 6.981 p = 0.0011, contralateral - F_3,30_ = 6.842 p = 0.0012, of treatment ipsilateral - F_1,10_ = 7. 424 p = 0.0214, contralateral - F_1,10_ = 4.981 p = 0.0497, and interaction ipsilateral - F_3,30_ = 5.599 p = 0.0036, contralateral - F_3,30_ = 4.01 p = 0.0164). To confirm that the observed changes to VFH stimulation were driven by inflammatory pain, we tested an additional 10 mice on the DPI paradigm and randomly gave half of the mice saline and the remaining half the commonly used anti-inflammatory drug meloxicam. We notice that at both time-points tested after DPI treatment, as predicted the vehicle only group developed a lowered mechanosensory threshold to orofacial VFH stimulation, while the analgesic group did not (Fig. 3E). These results are consistent with our DPI model evoking inflammation, and our read-outs being at least partially driven by inflammatory pain.

In regards to potential sex differences, none were found at baseline for any behaviors, based on t-test between females and males with Welch’s correction. For MGS, females were 0.2183 ± 0.06901 and males were 0.3533 ± 0.0726, (t = 1.348 df = 9.974, p = 0.2076). For the Von Frey transformed threshold, which will be discussed in detail in the next section, females were 1.027 ± 0.537 and males were −0.02641 ± 0.5075 (t = 1.426, df = 9.968, p = 0.1844). For the Von Frey Score, females were 1.522 ± 0.09181 and males were 1.311 ± 0.1824 (t = 1.031, df = 8.857, p = 0.3299). We conclude that at baseline there are no sex differences in our behavioral measurements, but this not preclude distinct behavioral responses or molecular mechanisms that drive pain hypersensitivity after dental pulp injury between the sexes.

Next, we analyzed our Von Frey data in two different ways guided by the response score and the “break point” built into the assay design. The apparatus allows the mouse to learn over time that their natural urge to explore may result in mechanical stimulation that could be painful, at which point they might choose not to expose their face and avoid stimulation, i.e. the stimulus at which this occurs is their “break point”. In the injured mice, we expect that this may occur with lower filament intensities over time, indicating allodynia. We also expect this may occur in the sham group at the higher stimulus intensities with repeated testing, which would indicate hyperalgesia. There is a large degree of disagreement in the field regarding what filament ranges constitute normally “painful” vs “non-painful” stimulation in the absence of injury or damage, which we have attempted to address for hind paw stimulation with VFHs^10^. Often this determination is made arbitrarily by the investigators based on human perception. Here, we use the Von Frey response scores and the break point to determine what range of filament weights correspond to a “non-painful” versus “painful” range. First, we examined the response scores by the weight of each VFH filament, to determine if they were higher across all filament weights, which would suggest the presence of both allodynia and hyperalgesia. Pulp exposed mice exhibit increasing response scores over time, particularly at lower filament weights (0.008–0.16 g) on both sides (Fig. 4A,C), which is not exhibited by the sham mice (Fig. 4B,D). This seems to indicate mechanical allodynia in the injured group. Because the mice reach the cut off score or begin to refuse stimulation, it is unclear if they also have mechanical hyperalgesia, but would be likely.

Second, we determined the weight of filaments that correspond to the break point (when the mouse takes more than 5 minutes to pass its face out of the opening) for both DPI and sham mice for each testing day. As time following pulp exposure increases, the intensity of the filaments where the mice indicate stimulation should stop becomes lower on both sides (Fig. 5A,C), such that by day 6 the break point occurs at 0.4 g ipsilateral and 0.6 g contralateral. In contrast, although there is some change in the number of sham mice that tolerate stimulation with filaments above 0.4 g after 7 tests, at least two mice tolerated the entire range of filaments during every test (Fig. 5B,D). Taken together this indicates that exposure of one tooth pulp results in a progressive development of mechanical allodynia, which is fully realized on day 4 post-exposure and increases in severity by day 6. The fact that we have bilateral effects following a unilateral injury likely indicates that central sensitization is manifest within this time frame.

## Discussion

In this study, we found that unilateral pulp exposure injury in mice to the first maxillary molar resulted in a statistically significant increase in MGS from the first 24 hours onward, which is taken as an indicator of spontaneous pain, but does not rule out a general increase in negative motivational affect. The pulp was still present at this time, but clearly exposed when examined histologically, supporting that the behavior could capture pain originating from the dental pulp, modeling pulpitis. Surprisingly, mechanical allodynia, as assessed by Von Frey filament testing, progressed more gradually, with initial changes in scores observed only on the contralateral side on day 2 post-injury, significant increases seen on both sides at day 4, and unwillingness to tolerate filaments above 0.6 g by day 6 post-injury. This work demonstrates that we have clear, easily identifiable behavioral readouts for trigeminal nociception in the mouse following dental injury. It is possible that the nocifensive behaviors and avoidance of stimulation captured by the score and break point in the Von Frey assay may not be due solely to pain that the mouse may be feeling, but could be related to anxiety or fear evoked during the testing conditions. Even if this is the case, there is clearly still a greater incidence of these behaviors in the injured mice as compared to sham controls. This is in line with other literature showing increased anxiety-like behavior and less food seeking in a high-risk environment after pulp exposure injury^8^. The fact that we see bilateral effects likely indicates that over the course of 4–6 days changes in the peripheral nervous system have led to central changes and sensitization. Associated with these behavioral changes, we observed significant increases in transcript levels of Tlr4, Trpv1, and Trpa1, but not Mrgprd, in the ipsilateral TG of injured mice as compared to controls at 24 hours post-injury. Taken together and in support of previous literature^7,28^, we believe these findings suggest that Tlr4, Trpv1, and Trpa1, may contribute to early changes resulting in the presentation of spontaneous pain, as indicated by the MGS. However, this does not rule out a role for Mrgprd in the progressive development of mechanical allodynia that seems to worsen around 4 days following pulp-exposure injury. Based on existing literature from other body regions^29,30^, TRPA1 could serve as a bridge between the early signaling indicated here and a hypothesized later process involving MRGPRD. TRPA1 is highly expressed in mechanosensitive dental pulp afferents^31^. How changes in the TG lead to central sensitization is not entirely clear, but could also be mediated by TRPA1. Several studies have demonstrated that application of mustard oil, the agonist of TRPA1, to the dental pulp produces nocifensive activity in the jaw muscle, due to central sensitization mediated by enhanced glutamatergic signaling in the medullary dorsal horn^32,33^. We speculate that prior to the onset of mechanical allodynia there may be paracrine signaling processes in the TG that amplify central input and contribute to the development of central sensitization.

Part of our objective in this study was to establish a time course of behavioral changes associated with tooth pulp exposure injury, which is considered by many to be a translationally relevant model for pulpitis^6,34^. To our knowledge, this is the first assessment of MGS following tooth pulp exposure injury, and somewhat surprisingly the first for facial Von Frey in mouse with this model as well. MGS and the rat equivalent RGS are significantly elevated following other types of dental pain, including tooth movement^35^ and mechanical load injury to the temporomandibular joint (TMJ)^36^, but in both of these cases, the elevation in score is transient, likely only corresponding with the presence of acute mechanical load. Our elevation in score does not subside, possibly due to the more invasive nature of the injury and the fact that ongoing inflammation is not being treated. In agreement with this idea, in a chronic model of trigeminal neuropathic pain, significant change in MGS is observed 10 days following the constriction injury^37^. It is possible, however, that there may be site or model specific differences. MGS was only transiently elevated following Complete Freund’s Adjuvant inflammation of the TMJ^38^, which does not have ongoing infection occurring in the model. In terms of change in the score, our data reflect a similar to slightly greater increase in MGS as compared to tooth movement^35^, and potentially within the lower end of ranges reported for an exogenous-CGRP migraine model^39^ and neuropathic injury of the infraorbital trigeminal nerve^37^. This indicates that the mice are likely in a level of discomfort or pain similar to other experimental pain states. Spontaneous pain is diagnostically associated with irreversible pulpitis, supporting the translational relevance of our findings^26^.

In addition to spontaneous pain, greater than 50% of patients with irreversible pulpitis also have mechanical allodynia with percussion of the tooth, and these patients have higher ratings of spontaneous pain than those without allodynia^26^. Facial Von Frey, an equivalent means of testing mechanical sensitivity in rodents is challenging, but not impossible in the mouse. Mechanical allodynia in the face has been examined in other experimental paradigms, but has not been published following tooth pulp exposure injury. Most studies have used rats as the model animal, and only one of these used the exact model we use here, where the pulp is left exposed and not treated with exogenous substances^34^. Our findings are in agreement with this previous work in rats. Tsuboi and colleagues also observed a reduction in threshold both ipsilateral and contralateral to the injury, first detectable at day 3, which worsened at day 5 and persisted at least 3 weeks later^34^. This period of time around day 3 or day 4 seems to mark a transitional state between the acute inflammatory response and development of pathological pain states often associated with chronic or ongoing pain. We speculate that the early change in MGS may be established by either the same or different mechanisms than those that produce mechanical allodynia later.

To begin to address this question, we examined the mRNA expression of Tlr4, trpv1, Trpa1, and Mrgprd using *in situ* hybridization at 24 hours following pulp exposure injury. A great deal of attention has been paid to TLR4 as a possible drug target for the treatment of inflammatory pain in various parts of the body^22^, but particularly in pulpitis given its role in recognizing molecular signals of bacterial presence and mechanical injury and upregulation in human pulpitis samples^21^. Furthermore, antagonism of TLR4 is associated with reversal of pain-associated behaviors in two different rat models of pulpitis^7,40^, including mechanical hypersensitivity in lightly anesthetized rats^40^. Our findings of increased Tlr4 in the trigeminal ganglia 24 hours following pulp injury suggest an association between the function of this receptor and at least increased malaise or spontaneous pain suggested by increased MGS. We need to directly antagonize TLR4 in the context of pulp-exposure injury to verify causality for increased MGS and determine if early intervention might prevent the delayed presentation of mechanical allodynia. It is possible that TLR4 upregulation begins a cascade of molecular events, as of yet not clearly identified, that establish a change in mechanical sensitivity.

Coinciding with the increase in TLR4 we also observed an increase in Trpv1 mRNA expression at 24 hours post injury, similar to increased protein expression found in rats with pulp exposure or CFA-induced pulpitis models^7,41^. Upregulation of the nociceptive channel TRPV1 has been demonstrated within 24 hours of LPS application to the tooth pulp, but returned to control levels 3 and 5 days later^18^. Furthermore, LPS can directly act on TRPV1+ trigeminal nociceptors via TLR4 signaling^23^. Antagonism of TRPV1 in the CFA model blocks mechanical hypersensitivity in lightly anesthetized rats^41^, suggesting that TRPV1 could be involved in the development of mechanical allodynia in our pulpitis model. However, given the delayed progression of mechanical allodynia reported here, it is likely that other events downstream of the increased TRPV1 expression in the ganglia are also involved in the pulp exposure model.

We also observed an increase in the expression of Trpa1 in the ipsilateral TG at 24 hours post-injury. As mentioned, targeting TRPA1 tooth pulp afferents, many of which are mechanosensitive^31^, can produce central sensitization^32,33^. Only one other study has examined protein expression following pulp exposure injury in rat molar^28^. They also observed increased expression of TRPA1, but it was not significant until Day 4^28^. Our differing results may be due to species differences, or may reflect a disconnect between the time to peak mRNA levels versus protein levels. Like TRPV1, there is also evidence for an interaction between TLR4 or LPS and TRPA1-related activity. In the DRG, there is evidence that TRPA1 is required for direct nociceptor responses to LPS, even in the absence of TLR4^19^. LPS increases the percentage of trigeminal neurons responding to the TRPA1 agonist acyl-isothiocyanate (AITC) as demonstrated by calcium imaging^24^. TRPA1 has been implicated in the development of mechanical allodynia in the lower body^42^, and is very clearly involved in central sensitization when TRPA1 is targeted in the dental pulp^32,33^, thus could be involved in the mechanical allodynia reported here.

While we observed increased expression of Tlr4, Trpv1, and Trpa1 24 hours post pulp exposure, we did not observe an increase in Mrgprd, also found in the pulp^17^ and directly implicate in cutaneous mechanical nociception^25,43^. However, this does not completely rule involvement of Mrgprd + trigeminal neurons in the development of delayed mechanical allodynia. Future studies will evaluate the expression of Mrgprd closer in time to the manifestation of mechanical allodynia around day 3 or 4. TRPA1 and Mrgprd have been linked in the development of mechanical allodynia in the lower body^29^, so some interaction could be involved in the dental pulp, particularly since TRPA1 is co-expressed in mechanosensitive dental pulp afferents^31^. Alternatively, paracrine signaling in the trigeminal ganglia via gap junction connections with satellite glia^40,41,44^ could allow for recruitment of the non-peptidergic TLR4 negative MRGPRD population by the peptidergic TLR4+/TRPV1 and/or TRPA1+ cell populations to contribute to central mechanisms underlying the mechanical allodynia we observed.

Although the model presented here is an inflammatory pain model, the DPI model has clear similarities to the orofacial mental nerve neuropathic (MNI) pain model, as we have previously described^11^. For example, we previously showed that both DPI and MNI induce trigeminal neuron expression of the injury gene activating transcription factor 3 (ATF3) and one week after both of these injuries we observed hypersensitivity to mechanical hind paw stimulation, indicating a central sensitization phenotype^11^. Although the studies here demonstrate that our methods are useful for assessing inflammatory pain in the tooth pulp, future studies are needed to determine if these methods will be useful in measuring pain in neuropathic pain models in the trigeminal region.

## Methods

### Animals

For behavior studies we used male and female adult wildtype mice consisting of a mixed CD1 and C57BL6/J background. Mice were 17–21 weeks old at the time of testing. 27 total mice were used in this study and purchased from the Jackson Laboratories and Charles River. We used 12 mice total in the initial behavior experiments with 6 males and 6 females, which allowed us to make the claims about no sex differences at baseline. For injury, we randomly separated the groups and 2/6 females and 4/6 males received the DPI surgery, while 4/6 females and 2/6 males received the sham surgery. To evaluate the effect of standard analgesia on the DPI model 10 additional male mice were used. Mice were maintained in a standard 12:12 light dark cycle (lights on at 07:00) tested within a time range of 08:30–13:00. Mice had access to food and water ad libitum when not being tested. All procedures were approved by the University of Pennsylvania Institutional Animal Care and Use Committee and follow the guidelines established by the National Institutes of Health.

### Dental pulp injury

Mice were anesthetized with ketamine/xylazine (i.p. 100 mg/kg and 12.5 mg/kg respectively) and positioned under a dissecting microscope and warming pad on their back, with their head supported at an angle, and their mouth propped open with forceps. After trimming the oral whiskers, the upper first maxillary molar was drilled on one side using ¼ round carbide burr until the enamel and dentin layers were breached and the pulp was exposed. This process took about 5 minutes. The enamel is hard and white, the dentin is gray, and when the pulp is visible vasculature and white to pink tissue can be seen in the hole in the enamel under the microscope. Sham animals underwent the same anesthesia, positioning and oral manipulation, but their teeth were not drilled. We provided moist food for the first 3 days following the procedure and monitored body weight daily during behavioral testing. The percentage of weight loss did not differ between the two groups at the beginning or at the end for the testing period (at 24 hours: DPI -2.9 ± 0.7% and Sham -3.5 ± 1.2% and at 6 days: DPI -3.3 ± 1.3% and Sham -3.8 ± 1.2%). Mice were either used for behavioral testing on days 1–6 post procedure (n = 6/treatment group), or were immediately euthanized on day 1 (n = 3 injured n = 2 naïve + 1 sham) to collect tissue. The same set of mice was used for Mouse Grimace Scale and Von Frey testing, performed on alternating days.

### Anti-inflammatory analgesic treatment

To evaluate alleviation of pain by standard analgesia, ten C57bl/6J males underwent the dental pulp injury procedure as described above. Half of the mice received meloxicam (s.c. 5 mg/kg) the day of the procedure and the next two days twice daily, while the other half received saline on the same schedule. These mice underwent behavioral testing in a treatment-blind manner, including Von Frey at two days post procedure (between drug treatments), and at three days post-procedure (following the end of drug treatments).

### Mouse grimace scale

The Mouse Grimace Scale is a scoring system developed in the laboratory of Jeff Mogil to objectively evaluate pain-like facial expressions following experimental procedures^13^, which has been adopted for many trigeminal pain models^36,37,39^, but not yet used to evaluate tooth pain in rodents. Mice (6/treatment group) were acclimated in the chambers at least twice prior to baseline testing, and were in the chamber for 10 minutes before recording began each day. Before the procedure and on days 1, 3, and 5 after injury, we video recorded mice for 10 minutes in clear acrylic chambers (4.3 W × 4.3 H × 11 L cm) on a mesh platform from the small end of the chamber with a camcorder (Sony, HVC) with digital zoom. A 3-way mirror was placed at the back of one end to facilitate assessment of unilateral grooming and to prevent the mouse from viewing the next acclimating mouse. From the 10-minute video, one still image for every second of video was extracted using Video to Picture Converter Software (Hootech). From these ~600 images, 10 were selected that contained a clear view of the animal’s face. All of the 480 selected baseline, sham, and post-pulp exposure images were cropped to show only the face and randomized for scoring in a Power Point file. Scoring of the images was performed blind to day and treatment, as indicated in the original method for 5 action units (orbital tightening, nose bulge, cheek bulge, ear position and whisker change), from 0 (not present) to 2 (very visible), and action units were averaged to arrive at the score for each image^13^. In some cases, the whiskers could not be viewed, so this unit was omitted for the score of that image. Performing the statistical analysis with or without the whisker change action unit did not affect the overall statistical results. For example images of a mouse before and after pulp exposure, see Fig. 2A,B.

### Mechanical allodynia assessment by von frey

For these studies we placed the animals in confined chambers with adjustable openings (Fig. 3). The mice were contained chamber about 7 cm in all directions, with an opening as wide as 2.5 cm. The animals were acclimated to the chambers once for 30 minutes the day prior to baseline testing. Their natural tendency is to put their face out of the opening when it is wide enough, but they are elevated from the floor, which prevents immediate escape. In this way, we can prompt the animal to present its face for stimulation. We then stimulated twice on either side of the face, alternating between sides, aiming for the region including the vibrissae to the point in front of the eyes. The animal’s response was scored from 0 to 4 based on early work in rats with neuropathic injury^15,45^ (see Table 1 for score description). We considered “threshold” to be the filament that either produced a score of 3 followed by a response of 2 or more, or the point the animal was no longer willing to pass its face out of the opening after about 5 minutes. The animals were tested on days 2, 4, and 6 post injury with the full filament series (Baseline Tactile Sensory Evaluators, consisting of 11 graded filaments from 0.008 g to 4 g).

**Table 1:** Score for Responses to Facial Von Frey.

Score	Response
0	no response
1	orientation to the stimulus or a slower head turn away from the stimulus
2	a rapid withdrawal that may or may not be followed by a single facial wipe
3	attacking or biting the filament or rapid withdrawal followed by 2 facial wipes
4	a rapid withdrawal with multiple facial wipes

### Preparation of tissue for histology

At 1 day post-injury, sham and naive, mice were deeply anesthetized with ketamine/xylazine and perfused with cold Phosphate Buffered Saline followed by 4% paraformaldehyde through the heart. We removed their trigeminal ganglia and either the remaining cranium or just the portion of the mouth containing the teeth and nerve roots. Trigeminal ganglia and mandibular regions were post fixed for up to 4 hours and overnight, respectively. Trigeminal ganglia were placed in 30% sucrose until they sunk (overnight), and then frozen in Neg50 media for cryosectioning (20 µm). Teeth were placed in 10% EDTA for approximately two weeks to decalcify, cryoprotected in 30% sucrose, embedded and cryosectioned (20 µm). All tissues were sectioned on a Leica cryostat onto superfrost plus slides, in a series of 16 (TGs) or 10 (teeth). Adjacent series of TG sections were selected for *in situ* hybridization using the RNAScope system for 2 probes. Four sections per left and right TGs from 3 animals were mounted on one slide. One series from the teeth underwent standard hematoxylin and eosin staining to visualize injury related alterations in the tissue.

### *In Situ* hybridization using RNAScope

Trigeminal ganglia were prepared using a modified version of the manufacturer’s recommendations for fixed frozen tissues used for fluorescence visualization. Briefly, slides were dehydrated in a graded series of alcohol, peroxidase activity was blocked with hydrogen peroxide, and protease IV was applied to the tissue for 30 minutes at room temperature before undergoing the RNAScope Multiplex Fluorescent v2 assay (ACD). The assay was performed according to the manufacturer’s protocol using two probes as we have previously described^46^. TG sections were assessed for overlap between Tlr4 (channel 1) and either Trpa1, Trpv1, or Mrgprd (channel 2). Channel 1 was visualized using opal dye 520 and channel 2 was visualized with opal dye 570 (1:1500 for both dyes). Tissues were imaged on a Leica SPE TCM using the same laser power and gain settings for all slides. Because we did not know the time course of pain changes in our DPI model, we opted to leave the pulp exposed, rather than applying a dye after exposure and sealing it and the injury site. Thus, we could not be fully certain that the neurons we visualized in the trigeminal ganglion came from the tooth pulp versus other trigeminal tissues. However, our own preliminary studies and others^16^ have shown that maxillary molar labeling with the DiI paste Neurotrace (Invitrogen) results in positive cells in all branches of the trigeminal nerve, therefore we imaged cell clusters observed in both the region where V3 and V2 meet, as well as the region where V1 and V2 meet, resulting in 2 images per section with the 20x objective. As we have previously described^46^ all cells with detectable signal were selected for quantification and the signal intensity of mRNA clusters observed within each cell was analyzed by drawing a region of interest around each cell and mean signal intensity in arbitrary units generated by ImageJ software was noted. The dimensions of the region of interest were kept constant throughout the analysis to avoid bias. This process was repeated for each channel including overlay images. The entire quantification was performed by an observer in a manner blinded to mRNA probes and channel assignments.

### Statistical analysis

Data were assessed for normality using the Shapiro-Wilkes test. Raw Von Frey thresholds were log-transformed to achieve normality so that parametric statistical tests could be used. For Von Frey and Grimace behaviors, two-way ANOVA with repeated measures and between subjects effects were used to determine if there were any significant effects of time, treatment, or a significant interaction, followed by with post-hoc Dunnett’s and Sidak tests where appropriate. For fluorescence intensity, an unpaired student’s t-test was used and **** represents a p-value < 0.0001. Statistical tests were performing using Graph Pad Prism (v8.3).
